# Supplementary material for: Light Response and Switching Behavior of Graphene Oxide Membranes Modified with Azobenzene Compounds
Source: Membranes (Basel). 2022 Nov 11;12(11):1131. doi: 10.3390/membranes12111131 (PMC9699301; doi:10.3390/membranes12111131)
Supplement: Supplementary file 1 [file membranes-12-01131-s001.zip › membranes-1964753-supplementary.pdf]

Light-Response and Switching Behavior of Graphene Oxide Membranes Modified with Azobenzene Compounds

Ilia Sadilov [1], Dmitrii Petukhov [2], Victor Brotsman [2], Alexandra Chumakova [3], Artem Eliseev [2] and Andrei Eliseev [1,2,*]

[1] Department of Materials Science, Lomonosov Moscow State University, 1-73 Leninskiye Gory, 119991 Moscow, Russia
[2] Department of Chemistry, Lomonosov Moscow State University, 1-3 Leninskiye Gory, 119991 Moscow, Russia
[3] European Synchrotron Radiation Facility, 71 Av. des Martyrs, F-38042 Grenoble, France
* Correspondence: author: eliseev@inorg.chem.msu.ru; Tel.: +7-(916)-954-60-41

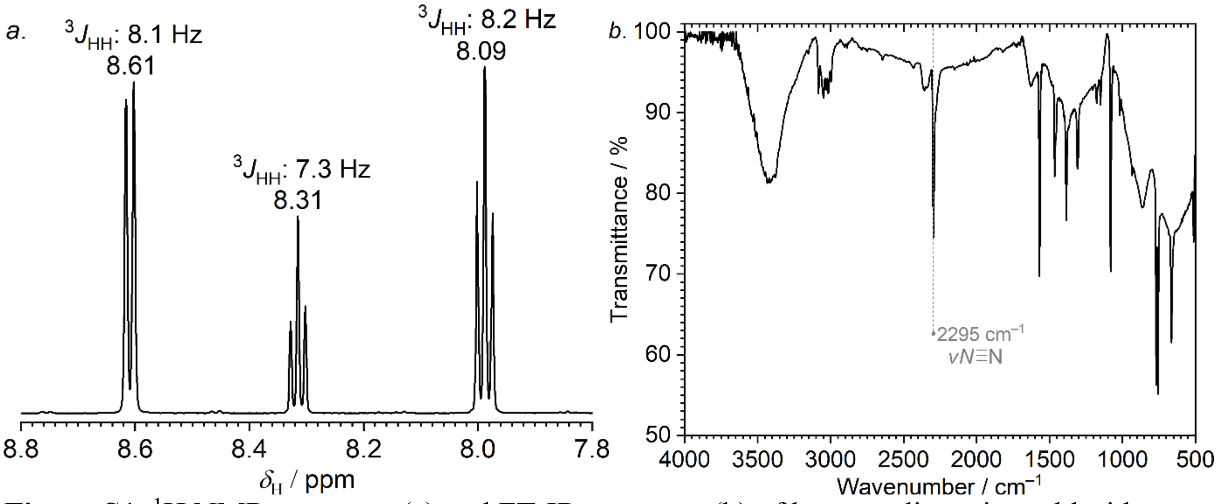

Figure S1. ^{1}H NMR spectrum (a) and FT-IR spectrum (b) of benzenediazonium chloride.

Benzenediazonium chloride. ^{1}H NMR (600 MHz, D_2O, ppm): δ_H 8.61 (d, $^3J_{HH}$ 8.1 Hz, 2H), 8.31 (t, $^3J_{HH}$ 7.3 Hz, 1H), 8.09 (t, $^3J_{HH}$ 8.2 Hz, 2H).

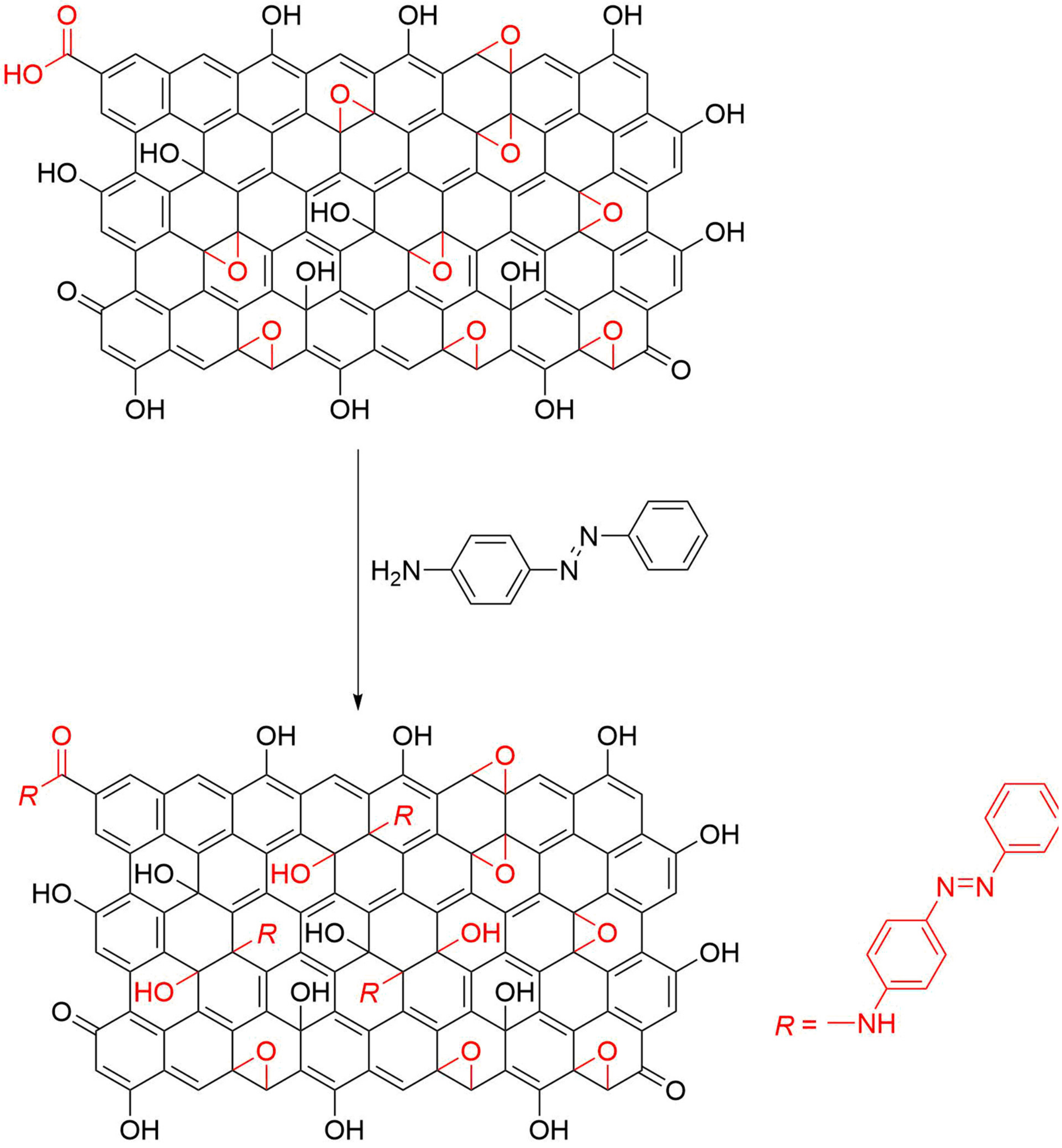

Figure S2. Reaction scheme of grafting pathways of *para*-aminoazobenzene onto GO nanoflakes.

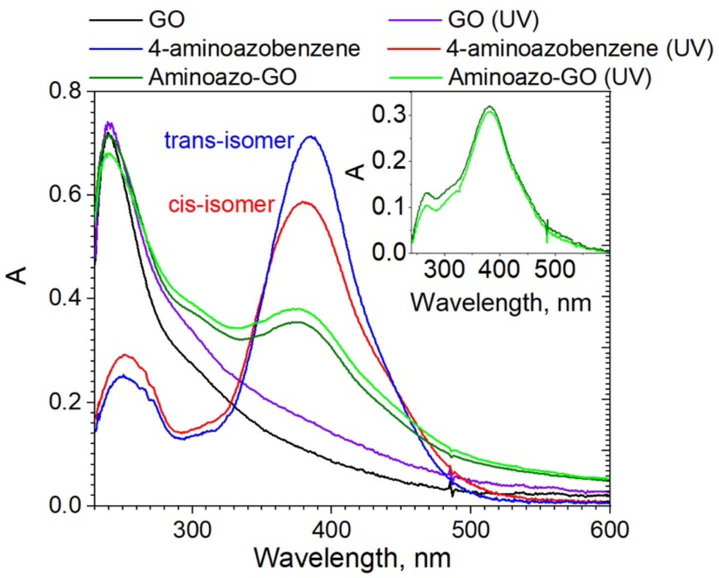

Figure S3. UV-vis optical absorption spectra of 4-aminoazobenzene, graphene oxide, aminoazo-GO prior and after UV exposure (325 nm, 10 mW) for 20 min. Inset graph shows difference spectra between aminoazo-GO and GO.

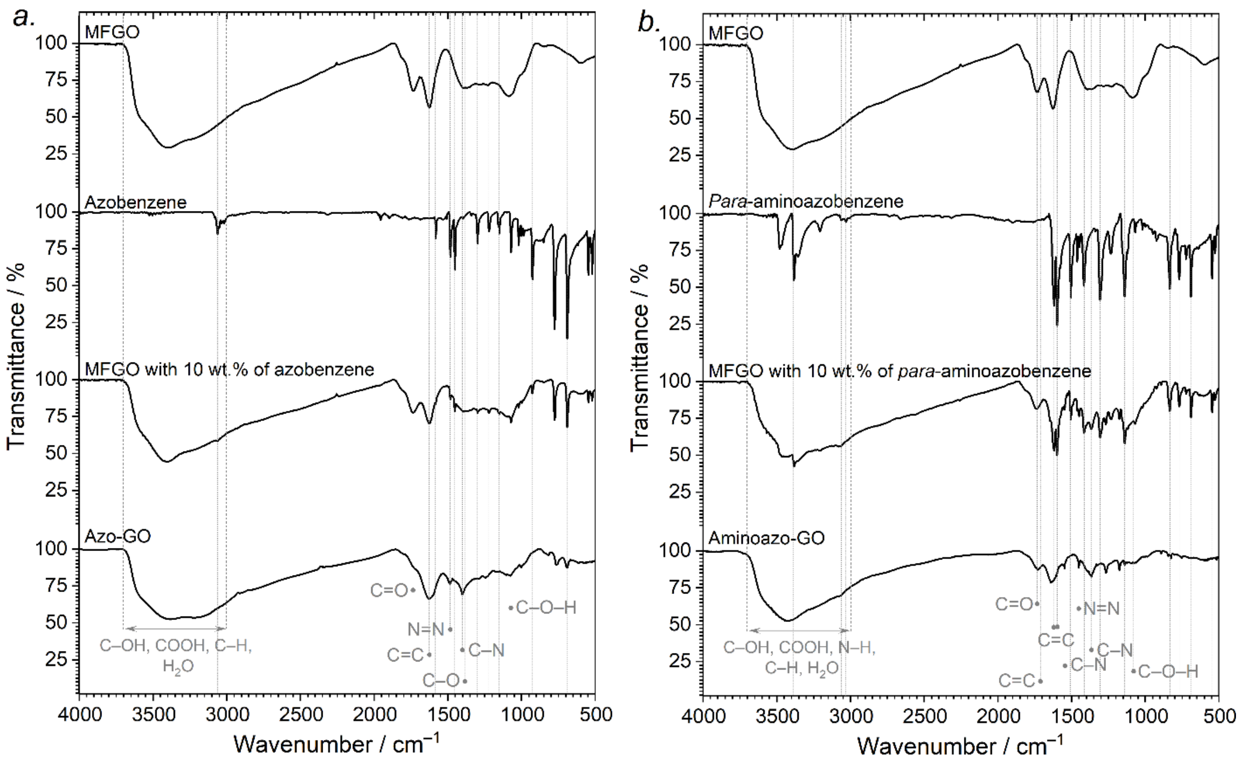

Figure S4. (a) FT-IR (KBr) spectra of azo-GO and (b) aminoazo-GO membranes in comparison with initial graphene oxide, initial azobenzene compounds and their control physical mixtures. The spectra reveal both shifting and broadening of azobenzene components signals in the membranes.

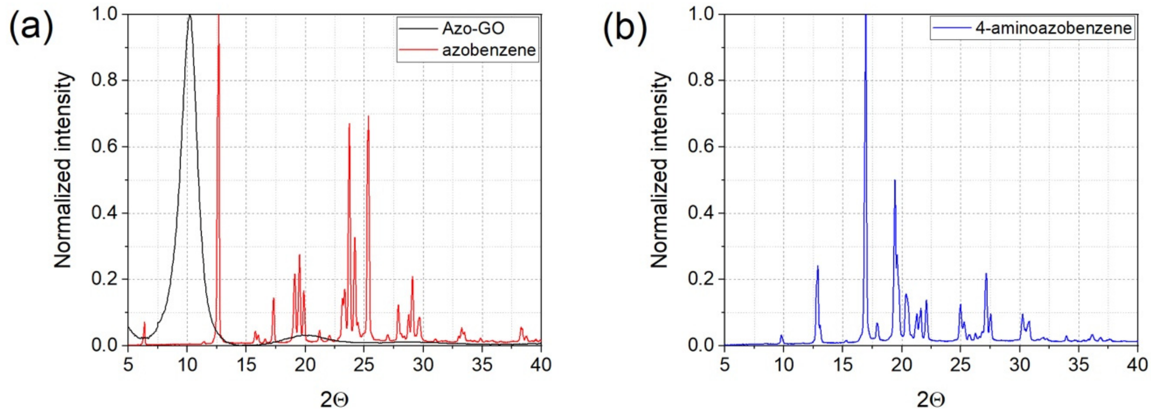

Figure S5. X-ray diffraction spectra of azobenezene (a) and 4-aminoazobenzene (b).

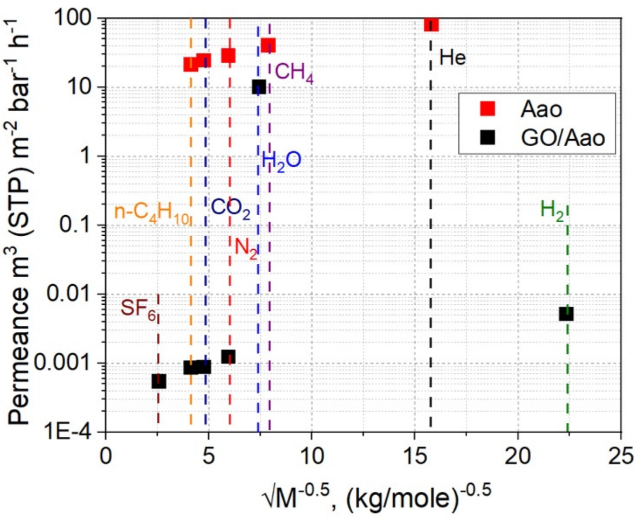

Figure S6. Gas permeance for initial anodic alumina membranes and GO/AAo composite membranes.

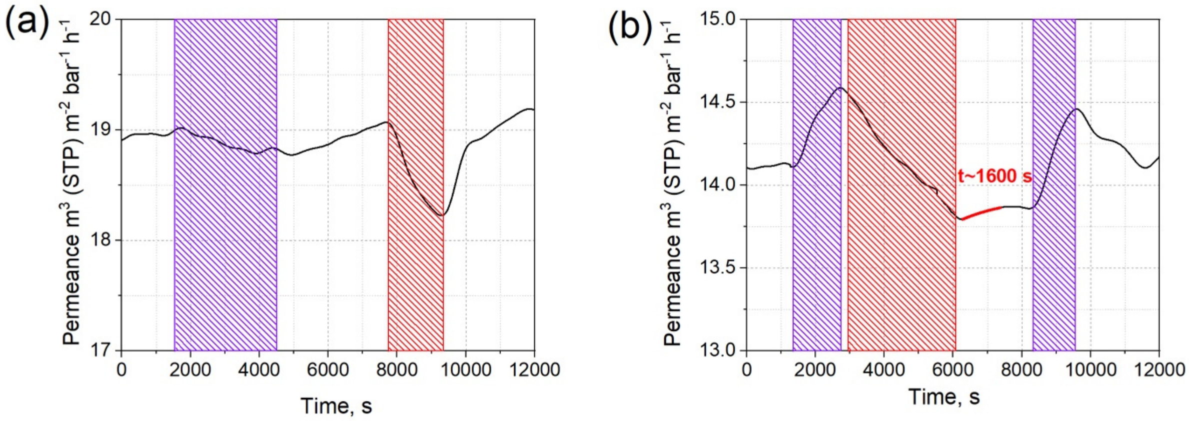

Figure S7. Variation of water vapor permeance for GO (a) and aminoazo-GO (b) under UV and IR irradiation.

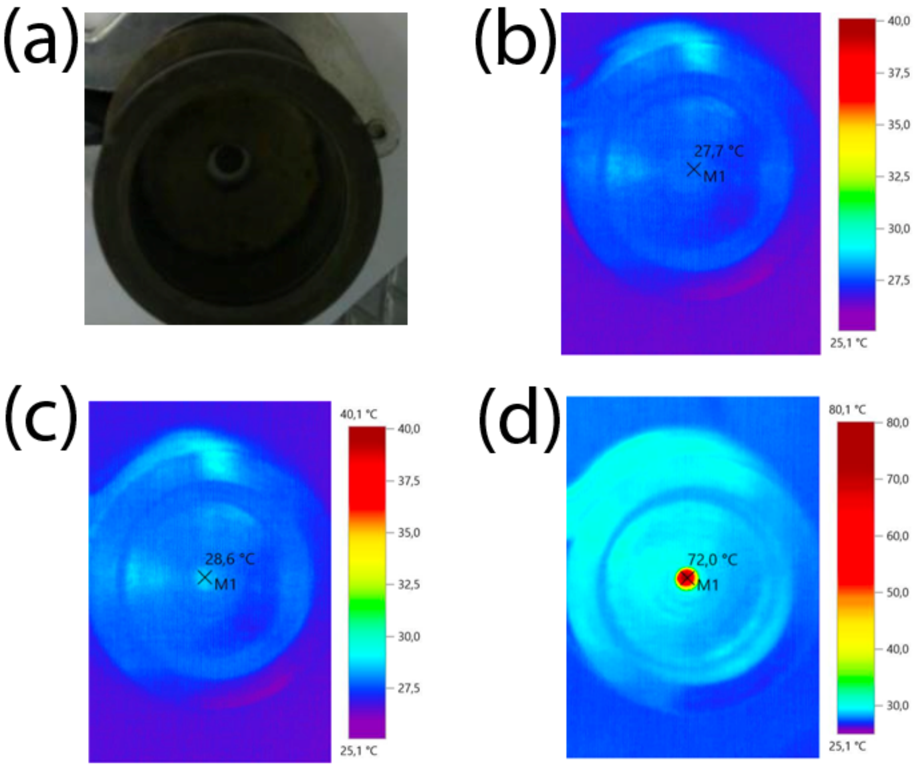

Figure S8. (a–d) Bottom-side IR- thermography maps of GO membrane fixed in the permeation cell, recorded under UV- and IR illumination of membranes. Point is the temperature of central point of the membrane.

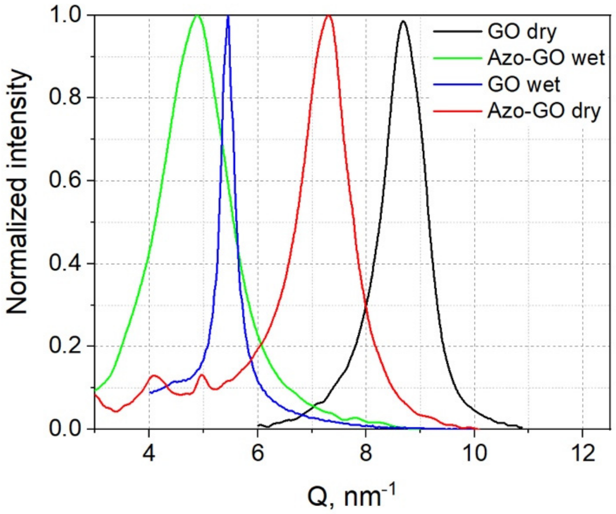

Figure S9. Comparison X-ray diffractions for GO and azo-GO in dry and wet states.

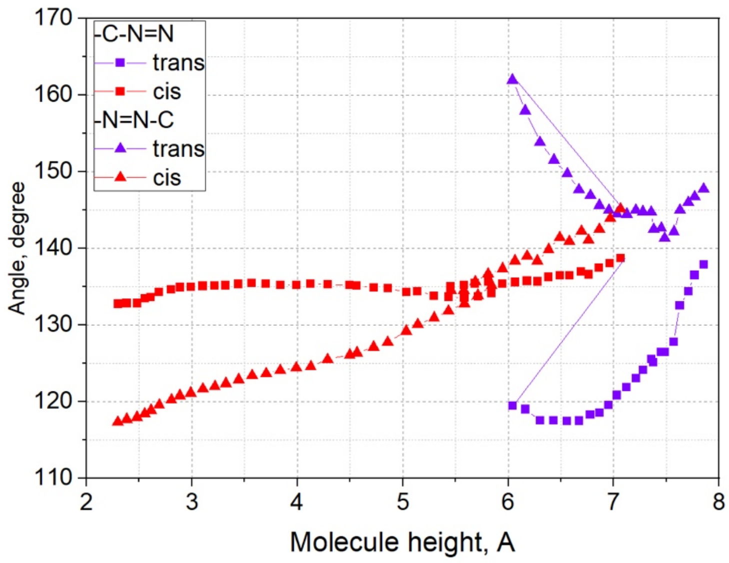

Figure S10. –C–N=N– and –N=N–C– angles for grafted azobenzene fragment into graphene oxide nanoflake.
